# Preparation for a potential outbreak of bluetongue virus in Ireland: surveillance design to estimate local prevalence after an initial case detection

**DOI:** 10.1186/s13620-025-00315-1

**Published:** 2025-11-20

**Authors:** Miriam Casey-Bryars, Jamie A. Tratalos, Jamie M. Madden, Guy McGrath

**Affiliations:** 1 Centre for Veterinary Epidemiology and Risk Analysis, School of Veterinary Medicine, University College Dublin, Dublin, Dublin 4 Ireland; 2 School of Veterinary Medicine, University College Dublin,Belfield, Dublin, D04 W6F6 Ireland

**Keywords:** Bluetongue, Surveillance, Prevalence, Ireland, Preparedness

## Abstract

Bluetongue virus serotype 3 emerged in northern Europe in 2023 and 2024. As of September 2025, Ireland is bluetongue free. However, to inform control decisions in the event of a possible incursion, a surveillance plan to detect cases and estimate prevalence is required. We created an active surveillance plan for 20 km radius temporary control zones (TCZs) after initial case detection. Potential TCZs (*n* = 1,062) covering Ireland were generated, and surveillance sample sizes were estimated based on cattle data in each TCZ. A two-stage (herd and animal level) design accounted for within-herd clustering. We simulated implementation of the surveillance plan in each TCZ to understand surveillance performance in the Irish cattle population. Within herd prevalence of 30% was used for all simulations, and we assumed the use of RT-PCR with perfect specificity and 99% sensitivity for diagnosis. Based on 100 simulations per TCZ, mean apparent animal level prevalence within tested infected herds ranged from 25 to 32%. With simulated between-herd prevalence of 5%, when between 39 and 61 herds per TCZ were sampled, apparent between-herd prevalence ranged between 3% and 6%. With simulated between-herd prevalence of 30%, and 10–11 herds per TCZ sampled, apparent between-herd prevalence ranged from 20 to 36%. Within herds, sampling 10–11 cattle was sufficient for prevalence estimation. We integrated Shiny and ArcGIS web applications to allow users to simulate scenarios under different settings. These include different test sensitivity and specificity, and different within- and between- herd prevalence contexts. This interface presents infected herds sampled, and true- and false- positives and negatives in a variety of conditions. Evidence from this scenario analysis can be integrated into a multi-pronged early warning and potential follow-up surveillance programme to facilitate decision making in the event of an incursion of BTV into Ireland. The URL for the web application associated with this paper is: https://www.arcgis.com/apps/dashboards/16722dde78d240f4a96303173bc6da2c.

**Supplementary Information:**

The online version contains supplementary material available at 10.1186/s13620-025-00315-1.

## Introduction

The emergence of bluetongue virus serotype 3 (BTV-3) in northern Europe has caused economic and animal welfare impacts throughout 2023 and 2024 [1–3]. Bluetongue (BT) is a disease of ruminants and new world camelids caused by infection with bluetongue virus (BTV), which is an *Orbivirus* from the *Reoviridae* family. It is primarily transmitted by midges of the genus *Culicoides*, but vertical transmission can also occur, as can transmission via needles or semen [4]. Evidence of introduction and spread of the BTV-3 was reported in England in 2023, and again since August 2024. As of September 2025, Ireland has escaped the spread of BTV in northern Europe. However, the potential for introduction and spread of midge borne infection has been evidenced by the emergence of Schmallenberg orthobunyavirus (SBV) in Ireland over the past decade [5–7].

BTV serotypes 1–24 are notifiable and considered category C diseases by the European Animal Health Law. This means that member states can implement an optional eradication programme recognised by the European Union (EU). The previous incursion of BTV-8 into northern Europe, beginning in 2006, had a large impact on the livestock industry. For example, between 2006 and 2018 in Germany, average direct and indirect costs relating to BTV-8 were €48.3 million and €132.1 million respectively [8]. For BTV-8 in the Netherlands, the cost of the 2006 epidemic was estimated at 32.4 million euro. The net costs of the BTV-8 epidemic of 2007 were valued at €164–175 million [9]. It was eventually eradicated in many countries by a comprehensive vaccination programme [10].

Evidence on the epidemiological and control features of BTV-3 in northern Europe is still emerging. There was extensive spread of BTV-3 in the Netherlands and surrounding countries in 2023 and 2024. The primary preventative measures focussed on emergency authorisation of an inactivated BTV-3 vaccine as well as biosecurity advice about indoor housing and ventilation to avoid midges [4]. Evidence is pending on whether BTV-3 vaccination will control the current outbreak in northern Europe, the outbreak impacts, and whether transmission will be as extensive in Britain as it has been in the other affected countries.

BTV-3 could be introduced into Ireland through (a) importation of an infected animal or foetus; (b) importation of infected biological material such as blood, or germinal products such as semen or embryos; or (c) windborne (or other) movement of midges bearing BTV-3 from an infected region. Since BTV-3 cases were detected in England in 2023, Ireland has suspended the imports of susceptible species from Great Britain due to the inability to meet import certification requirements. Germinal products may still be imported from Great Britain into Ireland and the EU, but only if the relevant animal health requirement for BT can be certified. The movement of susceptible animals and germinal products from Northern Ireland is permitted when carried out according to usual conditions. Under EU law, the movement of susceptible species and germinal products from EU countries is permitted, but it is not without risk and can only take place where detailed certification requirements can be met. However, given the evolving disease situation in Europe and the risk that purchased animals may not meet intra-EU movement certification criteria, the Department of Agriculture, Food and the Marine’s (DAFMs), National Disease Control Centre (NDCC) advises farmers to avoid these movements if at all possible [11]. Regardless of whether they are travelling from or through a country affected by BT, all ruminants and camelid animals originating from mainland Europe must be isolated on arrival and commence a programme of post-entry testing within 5 days of entry into Ireland, which is performed by DAFM [11].

The Hybrid Single Particle Lagrangian Integrated Trajectory (HYSPLIT) atmospheric dispersion model [12] can generate windborne particle trajectories. It is used in real-time to estimate the likelihood of windborne transfer of midges from a BTV infected area into Ireland, given weather conditions. DAFM uses this tool, described previously in the context of Schmallenberg virus incursion [6], to inform programmes of enhanced passive surveillance for clinical signs of BT.

In addition to passive surveillance, commonly used tests to diagnose BT include detection of viral RNA in whole blood samples by reverse transcriptase polymerase chain reaction RT-PCR [13, 14], detection of antibodies against BTV by serology [14], and adoption of the serology ELISA to test for antibodies in either individual or bulk milk samples [15]. Other more involved tests include virus isolation, genetic typing and sequencing, and virus neutralisation testing.

It is unknown how extensive BTV transmission in Ireland would be after an incursion. Effective surveillance to detect cases in a potential temporary control zone (TCZ) could assist Irish stakeholders in making informed decisions about control. Therefore, in this report, we explore a scenario where an incursion of BTV has already been detected, and detection of further cases in a 20 km radius TCZ around the initial case is required to estimate local prevalence. The purpose of such surveillance would be to enable an understanding of the extent of BTV transmission and to subsequently inform control measures.

## Methods

### Definitions

#### Sensitivity

The conditional probability of testing positive given the animal is truly infected. (Proportion of infected animals that test positive).

#### Specificity

The conditional probability of testing negative given the animal is truly uninfected. (Proportion of uninfected animals that test negative).

#### Between-herd prevalence

The proportion of infected herds (a herd is considered infected if it has at least one infected animal).

#### Within-herd prevalence

The proportion of infected animals within infected herds.

#### Apparent prevalence

The proportion of test positive units (animals or herds) out of all units tested.

#### Positive predictive value

The probability that an animal with a positive test result has the disease. This depends on test sensitivity, test specificity and disease prevalence.

#### Negative predictive value

The probability that an animal with a negative test result does not have the disease. This depends on test sensitivity, test specificity and disease prevalence.

### Surveillance design

Potential targeted surveillance design for a 20 km radius TCZ after the detection of an initial case of BTV-3 is described here. This surveillance would be in addition to (a) enhanced passive surveillance for clinical and postmortem signs of BT; (b) post import testing; and (c) targeted surveillance based on the likely sites of windborne midge dispersion. There is also the possibility for additional national surveillance approaches including bulk milk and syndromic surveillance in dairy herds. These additional surveillance approaches are not described here.

Annex V, Part II, Chap. 1, Sect. 4 of Commission Delegated Regulation (EU) 2020/689, a regulation supplementing the overarching European Animal Health Law (Regulation (EU) 2016/429), in the context of an approved eradication programme, describes the requirements for the recognition of the active surveillance for infection with BTV. These are based on geographical units of either grid squares of 45 km * 45 km but can be adapted to fit with geographical boundaries, such as counties or national administrative zones. These may include the TCZs planned by Ireland in the event of a BTV-3 outbreak. Surveillance must be carried out at least annually, or monthly during the season of vector activity where regular information is needed due to the risk of the infection spreading. The Irish vector active season is defined as from April to December in Ireland [16, 17].

According to EU legislation, the surveillance programme must have the capacity to detect, with a 95% level of confidence, infection with BTV with a target prevalence rate of 5%. This requirement does not distinguish between within- and between- herd prevalence. However, despite being a vector borne disease, herd level clustering of BT is recognised [18, 19]. A case study in France showed that an animal-level only surveillance programme, based on a 20% target overall animal level prevalence, may not have detected BTV-8 present between 2013 and 2015, and the programme was subsequently updated to consider herd level clustering and lower target prevalence [10]. Surveillance in England also took herd level clustering into account and translated the EU target of 5% overall animal-level prevalence into 50% between-herd prevalence and 10% within-herd prevalence [20]. These surveillance programmes face the challenge of expected low prevalence (requiring more sampling to detect cases) with the desire to limit the number of farms and animals that would need to be sampled due to resource constraints [10, 20].

For these reasons, we design a two stage (herd and animal level) surveillance plan, considering expected between-herd and within-herd prevalence of BTV-3. For logistical and testing cost reasons, we aim to minimise the numbers of herds requiring visits. Our geographical sampling unit is the 20 km radius TCZ.

### Expected prevalence in the absence of historical BTV prevalence data from Ireland

As Ireland has never had a BT outbreak, assumptions for prevalence are based on data from the BTV-8 outbreak in England in 2007 and 2008 [21] and on the recent BTV-3 outbreak in 2023 in the Netherlands [2, 15]. We supply a summary of these data in the Supplementary Material. These assumptions may be updated using our interactive application, as further BTV-3 data become available.

Transmission characteristics of BTV-3 may be different in Ireland due to temperature, weather, environmental, and livestock management conditions [10, 18–20]. Examples of potentially differing livestock management systems may include predominantly grass-based livestock production in the summer months [22], as well as an extensive cattle movement network [23]. The ecology and vector competence of the *Culicoides* species present in Ireland may also impact BT epidemiology [10, 16, 17]. Two surveys have confirmed that *Culicoides* species capable of transmitting BTV are present in Ireland [16, 17] but their competence for extensive transmission will also depend on climatic and environmental factors [19, 24, 25]. Based on English and Dutch data [2, 15, 21] we tentatively consider estimates of between-herd prevalence of 5% to be reasonable for an early stage BTV-3 spread, and 30% as BTV-3 is becoming established in a region. If considering 30% of animals in 5% of herds, the overall animal level prevalence is 1.5%, well within the EU target of 5% and closer to aligning with European Food Safety Authority (EFSA) advice to consider animal level prevalence as low as 1% [10]. As our understanding of expected BTV-3 prevalence in Ireland may evolve, or stakeholders may opt to use an alternative diagnostic test, we have built a web application to explore a range of expected within- and between-herd prevalences, as well as different diagnostic test sensitivity and specificity values (https://www.arcgis.com/apps/dashboards/16722dde78d240f4a96303173bc6da2c).

### Irish cattle data

Herd locations were derived from computing the centroid of the largest fragment of land for each farm based on DAFM’s Land Parcel Information System (LPIS) for herds registered in 2024 [26]. Herd size was estimated from the number of animals per herd subjected to a bTB test in 2024 and filtered to remove repeat tests. These data were extracted from DAFM’s Animal Health Computer System (AHCS).

### Definition of TCZs

**Fig. 1:**
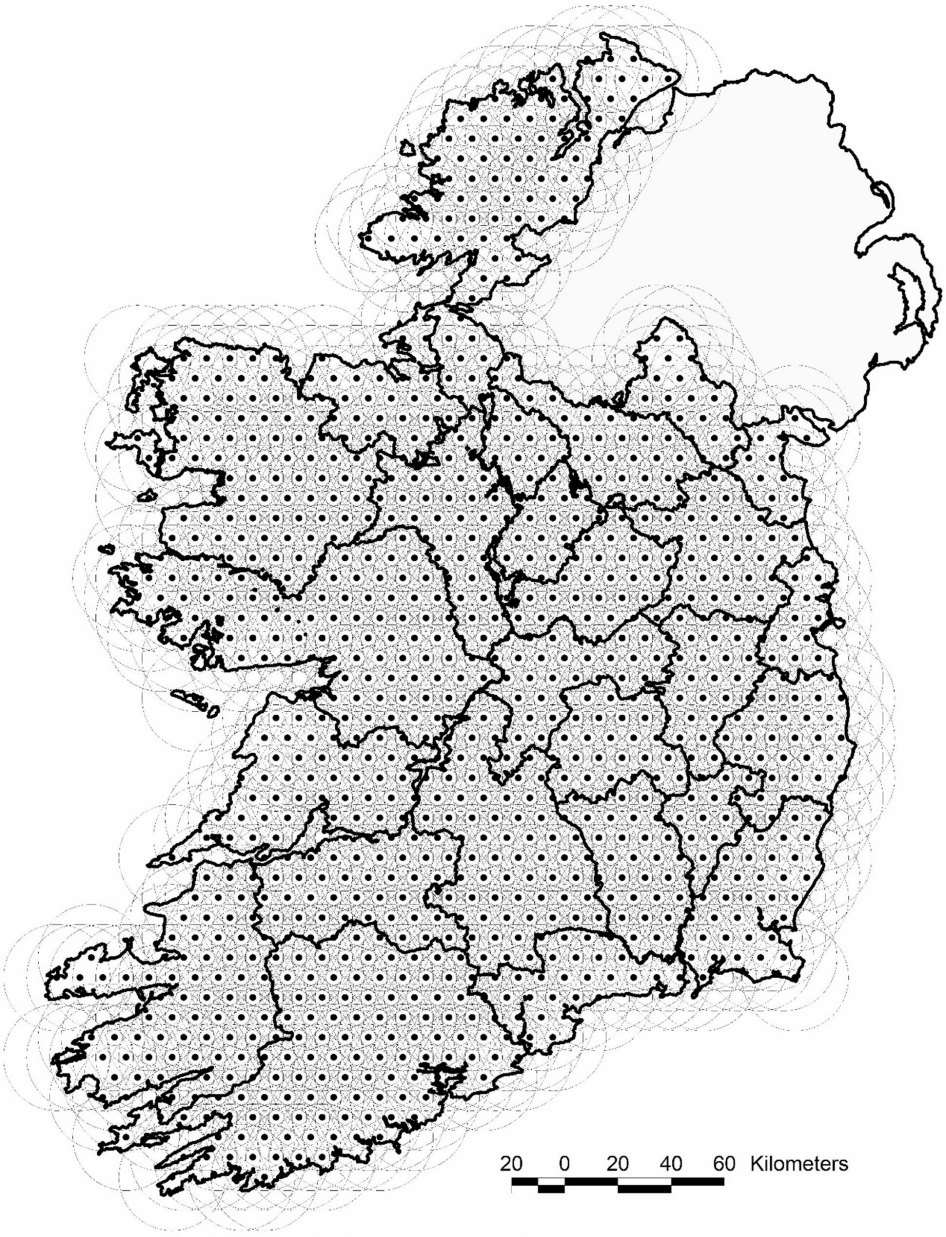
Hypothetical 20 km radius temporary control zones (TCZs)

Overlapping circles (*N* = 1064) with a radius of 20 km were designed to cover the island of Ireland, representing hypothetical TCZs (Fig. 1). Based on the herd centroids, a list of all herds and herd sizes in each TCZ was generated for use in sample size estimation and scenario exploration. Having overlapping zones allows for estimates to be generated in the zone with its centre closest to an area of interest.

### Species selection

We considered cattle surveillance to better enable case detection and made our estimates based on sampling this species only. Cattle attract more midges than sheep [10, 27, 28] and within-herd apparent prevalence has been found to be higher in cattle than sheep [2]. A comprehensive review by EFSA estimated that live BTV could also be isolated for a longer period from cattle (75th percentile 50 days for cattle compared to 30 days for sheep), whilst suggesting a detection window of 4–5 months for detection of BTV nucleic acid by RT-PCR in both species [10]. Reports on the BTV-3 epidemic in the Netherlands suggest a similar RT-PCR detection window, describing PCR positivity in cattle 180 days after infection, compared to 100 days in sheep [4].

Supplementary Fig. 1, adapted from Tratalos et al. (2020) [23], summarises the Irish cattle population in terms of herd and cattle density as well as herd sizes and the distribution of dairy and beef management types. Larger cattle herds, and higher densities of cattle are present in the South and the East of Ireland. Based on experiences with Schmallenberg virus (Supplementary Figs. 2 and 3, as previously reported by McGrath et al. (2018) [6]), the South and East may also be the areas with highest likelihood of a BTV incursion via long-distance windborne midge movements and of further transmission. Supplementary Fig. 4 shows that, in contrast to cattle, the Irish sheep population density is highest in higher altitude areas [29].

### Testing for evidence of bluetongue virus infection

#### Diagnostic characteristics for RT-PCR

For our main text, we assume that animals selected for testing are tested with a real-time RT-PCR to detect BTV genome [13]. Similarly to Grace et al. (2017), we assumed a sensitivity of 99% and a specificity of 100% [20].

Vandenbussche et al. (2008) [30] reported a diagnostic sensitivity and specificity for the real time RT-PCR of 99.5% (95% CI: 99.0–100) and 98.5% (97.1–100), respectively. The EFSA opinion on these figures highlighted that, when testing large numbers of negative animals, for example in the early stages of an outbreak, false positive test results may be observed. However, in this context, specificity can be increased if positive results are subsequently tested by a serotype specific PCR [14].

We chose to conduct the simulations reported in our main text assuming perfect test specificity. This is because we believe the serial testing approach to maximise specificity, as advocated by EFSA, would be used in the context of the early stages of a BT outbreak in Ireland. However, we report a lower (99%) specificity scenario in our Supplementary Results, which highlights false positive issues in the context of low prevalence and imperfect specificity, if interpreted without epidemiological context. All assumptions relating to prevalence and diagnostic test characteristics for our series of scenarios are defined in Table 1 in the results section. Furthermore, the reader can explore the impact of a range of specificities, sensitivities and prevalences on false positive and negative test results using the interactive web application associated with this paper.

**Table 1:** Our assumptions about true prevalence of infection between and within herds, diagnostic test sensitivity and specificity and related estimates of positive and negative predictive value (PPV and NPV)

Scenario	Between herd prevalence	Within herd prevalence	Test sensitivity	Test specificity	PPV	NPV
Main text lower prevalence	5%	30%	99%	100%	100%	99.98%
Main text higher prevalence	30%	30%	99%	100%	100%	99.90%
Supplement lower prevalence	5%	30%	100%	99%	60.36%	100%
Supplement higher prevalence	30%	30%	100%	99%	90.81%	100%
cELISA PPV and NPV calculation lower prevalence	5%	30%	89.2%	98.4%	45.92%	99.83%
cELISA PPV and NPV calculation higher prevalence	30%	30%	89.2%	98.4%	84.65%	98.92%
cELISA PPV and NPV calculation highest prevalence	50%	50%	89.2%	98.4%	94.89%	96.47%

#### Diagnostic characteristics for competitive ELISA

A competitive ELISA (cELISA) to detect antibodies against BTV (ID SCREEN^®^ BLUETONGUE COMPETITION^1^) is also available in Ireland. An EFSA review of papers published up to 2016, estimated cELISA sensitivity in cattle of 89.2% (range 83.0% − 100%) and specificity of 98.4% (range 95.8%–99.5%). We report positive and negative predictive values based on EFSA reported cELISA diagnostic characteristics, but do not report full simulation results, as the lower specificity may not be appropriate for the low prevalence/early-stage outbreak context, given the likelihood of false positive results.

#### Web application to explore alternative scenarios

To allow exploration of a range of scenarios with different between- and within- herd prevalences, and for alternative diagnostic test sensitivities and specificities, we translated our simulation code into a Shiny web application [31]. We used an ArcGIS dashboard [32] to present our TCZs and integrated both applications to be presented alongside each other online. Users can select a 20 km radius circle and model surveillance outcomes. These include infected herds tested, true and false positive herds, false negative herds, as well as a range of other measures. The joint application can be accessed here:

https://www.arcgis.com/apps/dashboards/16722dde78d240f4a96303173bc6da2c.

### Sampling logistics

We sought to minimise the number of farms visited and did not limit how many animals within the herd were required to be sampled.

### Further targeting

Larger cattle herds were more likely to test positive for BT in the English 2007–2008 outbreak [21]. At animal level, risk factor studies in Pakistan and South Korea reported either no effect [33] or a protective effect [34] of increasing herd size on the probability of an individual animal testing positive. We selected herds of greater than 100 cattle in size. A previous BT survey in England also focussed on cattle herds with a similar rationale as ours, relating to greater attractiveness of cattle to midges (compared to sheep) and importance for virus transmission [20]. Grass based cattle production predominates in Ireland from Spring to Autumn [22].). Because of this, few herds are managed indoors, and we assumed that the majority of cattle herds would be at pasture at dawn and dusk during the grazing season. Supplementary Figs. 1–3, adapted from Tratalos et al. (2020) [23] and McGrath et al. (2018) [6], show that the largest cattle herds and highest cattle densities are in the South and East of Ireland, which were the areas of the previous Schmallenberg virus incursion and transmission.

Our choice of 20 km TCZ based geographical surveillance units is supported by the literature on BTV transmission distances and *Culicoides* biology. A previous study of insect vector dispersal stratified it into (a) short-distance movements, independent of wind, and (b) long-distance wind-aided migratory movements [35]. Hendrickx et al. (2008), in their analysis of the BT epidemic in northern Europe in 2006, estimated that half of the new weekly cases were distributed within 5 km of the closest case reported in the previous week, and 95% of new cases were distributed within 31 km [36]. Using data from Germany, France, Belgium, the Netherlands and Luxembourg, Sedda et al. (2012) similarly estimated that 54% of outbreaks occurred through transmission of BTV within 5 km or less, and 92% of outbreaks were due to transmission within 31 km. No differences between upwind or downwind movement of infection were reported [37]. Gubbins et al. (2014), using data from Belgium and the Netherlands, predicted regional spread of BTV with a mean radius of 23.2 km with no movement restrictions, and 9.4 km with movement restrictions [24]. These data suggest that the planned 20 km radius TCZ around the first case identified is a reasonable geographical unit for surveillance. Separately, and outside the remit of this current paper, the risk of rarer longer distance wind-associated midge migrations, as described by Hendrickx et al. (2008) [36], can be assessed using the HYSPLIT wind-dispersion modelling tool [6].

### Sample size estimation

We used the “EpiR” package [38] to estimate the sample size required in each TCZ. The two-stage representative survey design tool in the “EpiR” package allowed consideration of both within- and between- herd prevalence. We calculated how many herds and how many animals from within each herd need to be sampled to be 95% confident of detecting disease at the herd and individual animal level based on our summaries of English and Dutch data (Supplementary Materials). We consider estimates of between-herd prevalence of 5% to be reasonable for an early stage BTV-3 spread, and, conservatively, 30% if BTV-3 has become established in a region. Within-herd prevalence of 30% was assumed.

Scenario simulation to explore effectiveness of surveillance.

To explore the effectiveness of our planned surveillance, including levels of false negative and false positive results under different test interpretation conditions, we conducted a simulation study. Static between- and within herd prevalence was assumed. The steps in the simulation were as follows.

i. For each TCZ, infected herds were simulated based on the expected between herd prevalence and the total count of cattle herds.
ii. For each potential TCZ, herds with 100 cattle or more were selected.
iii. The number of herds required to be sampled, based on the “EpiR” based sample size calculation was randomly selected from the herds defined in point (ii).
iv. If any of the selected herds had been simulated as infected herds, infected animals within that herd were simulated based on expected within herd prevalence and herd size.
v. From each selected herd, the count of animals required to be sampled, based on sample size calculation, was randomly selected.
vi. Test results in selected animals were simulated based on the animal’s simulated true infection status, and test sensitivity and specificity.
vii. Simulated true and apparent prevalence was compared, and the levels of false positives and false negatives under different test and prevalence conditions were reviewed.

Our simulation code and an example subset of data are available here: https://github.com/miriamcasey/BTV_surveillance.

### Software

Data processing was performed in Microsoft SQL Server 2012. The GIS software package ArcGIS Pro 3.3.0 [39] was used to generate the 20 km buffers representing the TCZs and to assign intersecting herds to them. All other analyses were conducted in the R statistical environment [40] and as previously highlighted, the “EpiR” package [38] was used for sample size calculations. Web interfaces were built using Shiny [31] for simulations and the ArcGIS Dashboard [32] for the TCZ map. These were integrated so that the TCZ map and the simulation tool were visible side-by-side to users.

## Results

### Results overview

Table 1 summarises the assumptions we made for analyses relating to between- and within- herd prevalence and diagnostic test characteristics. We also report the positive and negative predictive values associated with them associated with each set of assumptions on Table 1.

### Population summary

6,852,137 cattle in 101,355 herds were present. Herd size ranged from a single bovine to 2,654 cattle, with a mean of 68, a median of 36 and an interquartile range (IQR) of 16 to 82. More than 100 cattle were present in 19.9% of the herds.

In the 1,064 hypothetical 20 km radius TCZs herd counts ranged from 68 to 3,548 (median = 1,658, IQR = 1,125–2,115), and cattle counts ranged from 1,372 to 242,297 (median = 120,536, IQR = 65,162–158,920).

### Exploration of positive and negative predictive values with different animal-level disease prevalence

#### RT-PCR testing positive and negative predictive values

A 5% between-herd prevalence and a 30% within-herd prevalence is consistent with a 1.5% overall animal level prevalence. A 30% between-herd prevalence and a 30% within-herd prevalence is consistent with a 9% overall animal prevalence. In our main scenario, we assume perfect diagnostic test specificity and therefore perfect Positive Predictive Value (PPV). Given our sensitivity of 99% and maximum 9% overall animal level prevalence, the negative predictive value (NPV) was also almost perfect (Table 1).

#### Competitive ELISA positive and negative predictive values

Our web-application can demonstrate how PPV and NPV decline in some prevalence and diagnostic test scenarios. For example, EFSA reported specificity of 98.4% for the cELISA [14] which would be associated with high levels of false positives in a low prevalence context. With a specificity of 98.4% and a sensitivity of 89.2%, the PPV is 45.9% for an overall animal level prevalence of 1.5% (corresponding to between-herd prevalence of 5% and within-herd prevalence of 30%, Table 1). However, if the prevalence increased to levels seen in the Netherlands’ BTV-3 epidemic, false-positives would decrease. For example, with 50% between and within-herd prevalence, the PPV, based on EFSA reported cELISA sensitivity and specificity estimates, is 94.9%. With 5% between-herd prevalence and 30% within-herd prevalence, NPV related to the EFSA reported cELISA diagnostic characteristics was 99.8%. With the 50% between and within herd prevalence cELISA NPV was 96.4%.

#### Sample size estimation for RT-PCR

Using the “EpiR” function for two stage surveillance design (“rsu.ssep.rs2st”), for a between-herd prevalence of 5%, and a within-herd prevalence of 30%, unspecified large herd and animal population sizes, 95% confidence of detecting disease at the herd and individual animal level, the sample size required was 62 herds per TCZ with 9 randomly selected animals per herd sampled. When between- and within- herd prevalence were set to 30%, 9 herds and 9 animals per herd were required to be sampled.

**Fig. 2:**
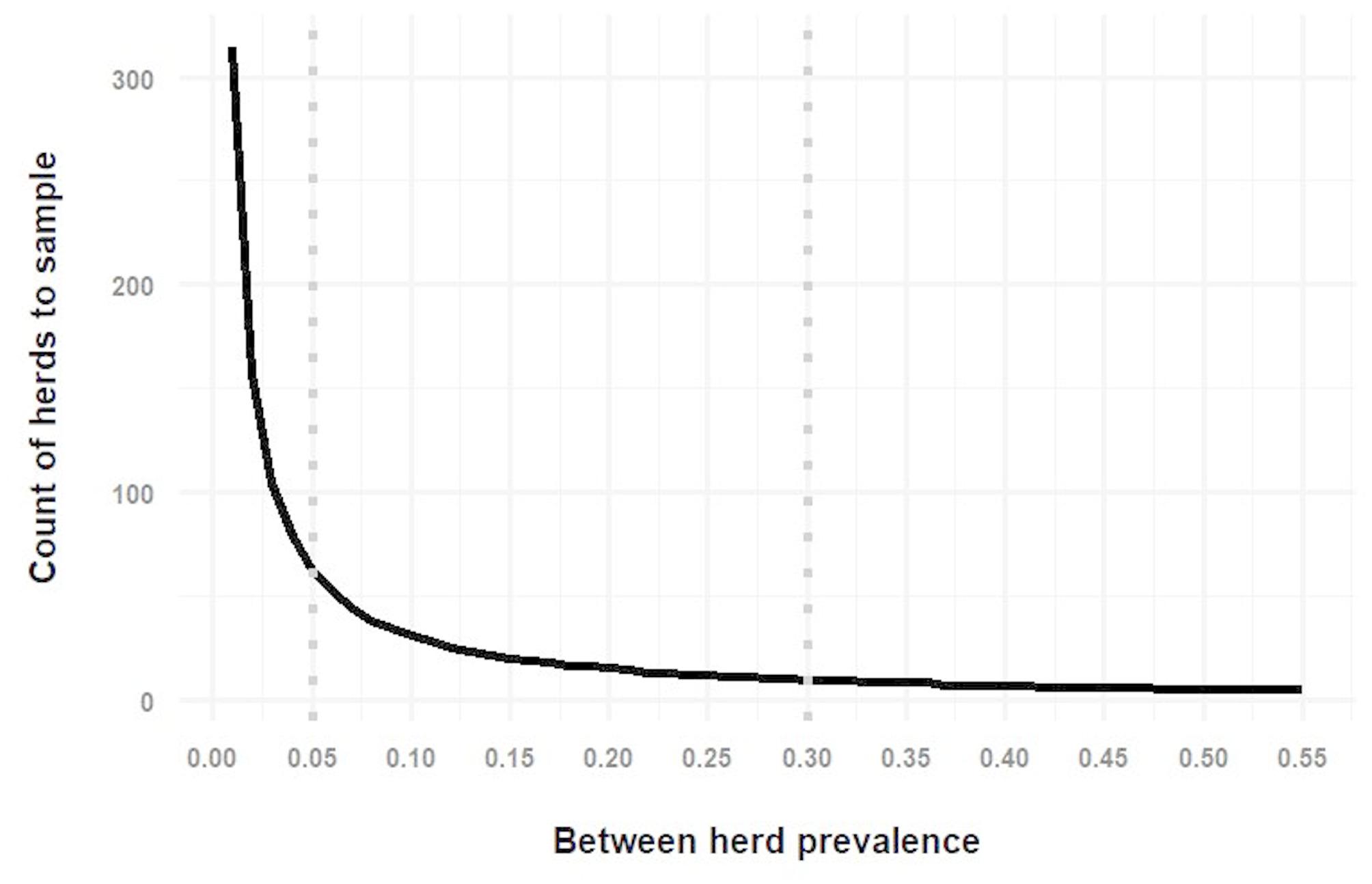
Count of herds required to be tested to detect a range of between herd prevalences. The vertical dotted lines represent between herd prevalences of 5% and 30% respectively

**Fig. 3:**
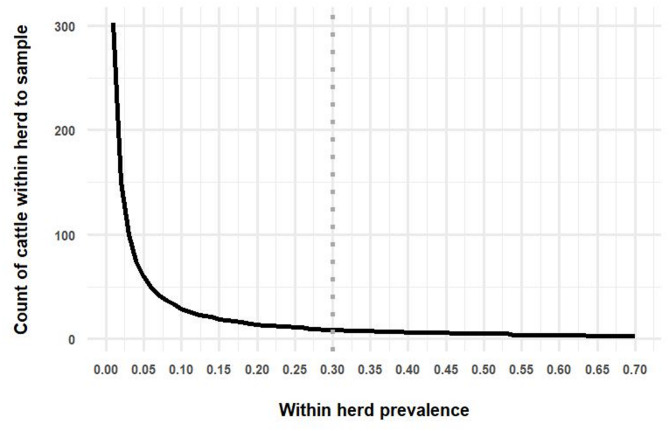
Count of cattle within selected herds required to be tested to detect a range of within herd prevalences. The vertical dotted line represents within herd prevalence of 30%

Figure 2 shows the count of herds to be tested given a range of between herd prevalences. Figure 3 shows the count of cattle within selected herds to be tested, given a range of within-herd prevalences.

When specific lists of herd sizes of herds containing more than 100 cattle each of the 862 selected TCZs were inputted into the “EpiR” tool, with between-herd prevalence of 5%, between 39 and 61 herds were required to be sample per TCZ. The sample size required varied in different TCZs depending on the total count of herds in the TCZ. For a between-herd prevalence of 30%, a sample size of between 10 and 11 herds per TCZ was required. With both scenarios, the within-herd prevalence was set at 30%, and between 10 and 11 cattle per selected herd were required for sampling.

#### Scenario simulation to explore the effectiveness of surveillance with 5% between herd prevalence

Because our surveillance design was focussed on herds with 100 cattle or more, and to enable a standardised approach for each TCZ, we selected these TCZs (> 70 herds with ≥ 100 cattle) for our simulation to explore the application of the sample size estimation to Irish TCZs. The count of herds in these selected TCZs ranged from 203 to 2,489.

We report simulation results for both 5% and 30% between-herd prevalence scenarios as median measures from 100 simulations for each TCZ.

Simulated between-herd and within-herd prevalence were 5% and 30% respectively, as expected. Based on this and the total herd count, between 10 and 127 herds per TCZ were infected. The number of herds tested per TCZ (based on inputting the list of herds and herd sizes from each TCZ into the “EpiR” tool) ranged from 39 to 61 and a median of between 1 and 3 infected herds were sampled per TCZ.

**Fig. 4:**
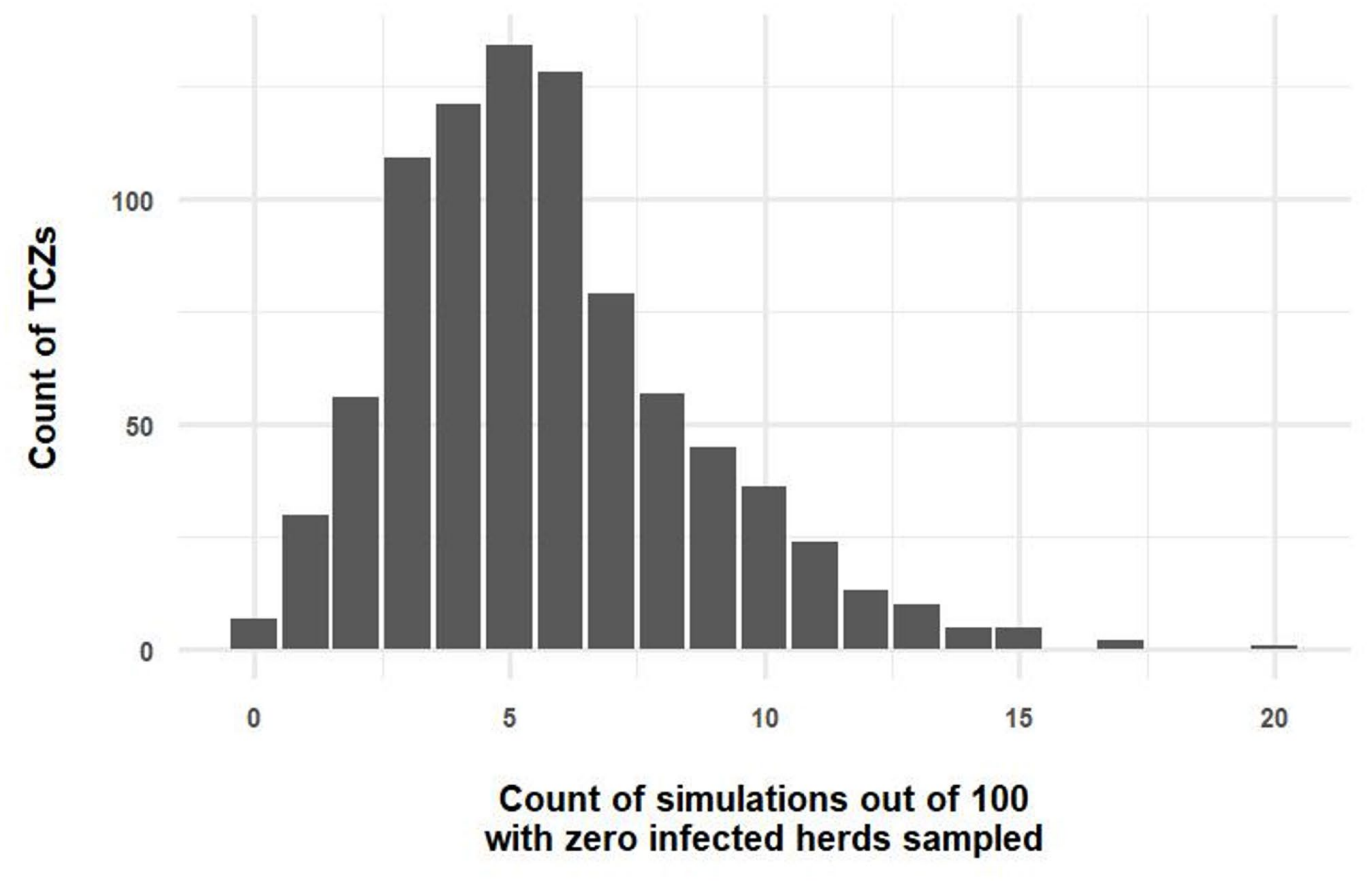
A bar-plot showing counts of simulations out of 100 replicates which had zero infected herds sampled within the temporary control zone (TCZ) on the x-axis and count of TCZs (out of 862) on the y-axis. Simulated true between-herd prevalence was set at 5%

Amongst all the repeat simulation experiments, of the 862 TCZs, few had infected herds sampled in all 100 repeat simulations. The median proportion of simulations with zero infected herds sampled was 5% (interquartile range (IQR) 4% – 8%) (Fig. 4).

Overall, simulation outputs aligned with “EpiR” tool calculations, with the surveillance programme detecting the expected between- and within- herd prevalence.

**Fig. 5:**
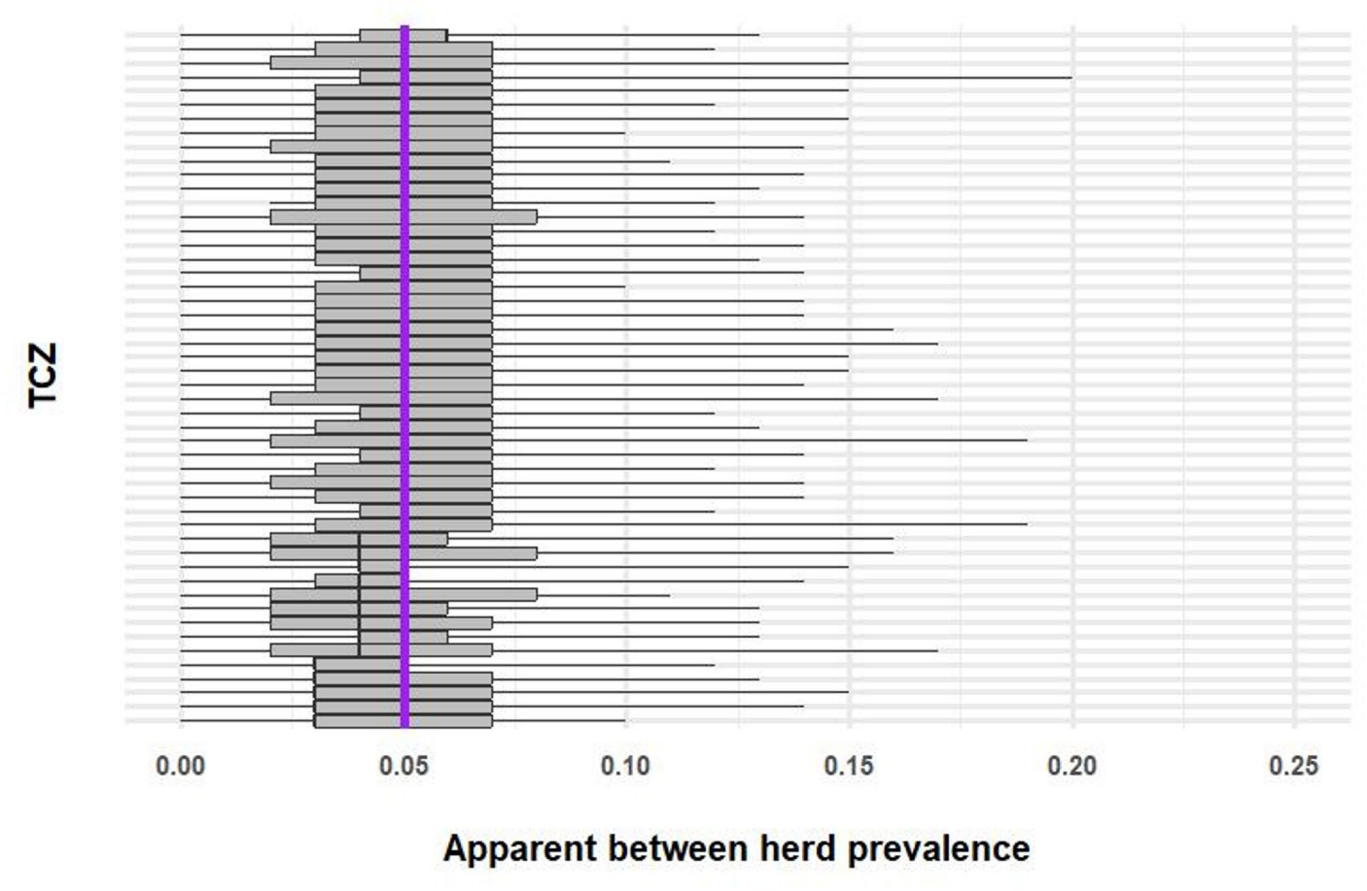
Apparent between herd prevalence in 50 randomly selected temporary control zones (TCZs) and simulated true between herd prevalence of 5% (purple line). The ends of the whiskers show the maximum and minimum, the box shows the interquartile range. The plot is ordered by median apparent herd prevalence. Only a sample of TCZs are shown, to improve plot clarity

Amongst the cattle selected for testing, median counts of test positive infected cattle (true positives) in each TCZ ranged from 4 to 10 and counts of test negative uninfected cattle ranged from 385 to 604. There were no false positive or false negative results simulated. Within test positive herds, median apparent animal level prevalence (based on median estimates from 100 simulations per TCZ) ranged from 25 to 32%. Apparent herd level prevalence was between 3% and 6% (Fig. 5).

#### Scenario simulation to explore effectiveness of surveillance with 30% between herd prevalence

**Fig. 6:**
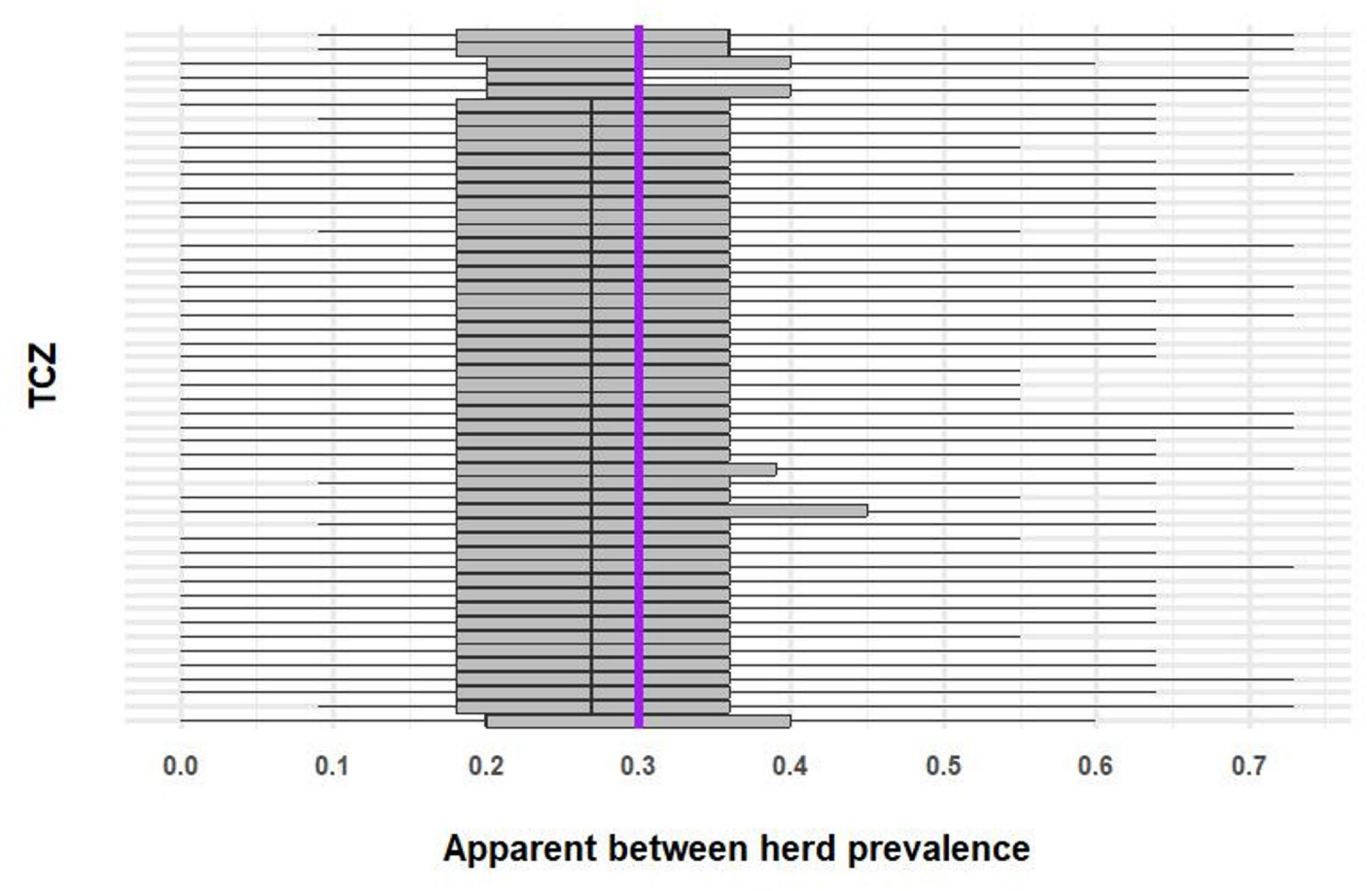
Apparent between herd prevalence in 50 randomly selected temporary control zones (TCZs) and simulated true between herd prevalence of 30% (purple line). The ends of the whiskers show the maximum and minimum, the box shows the interquartile range. The plot is ordered by median apparent herd prevalence. Only a sample of TCZs are shown, to improve plot clarity

When between- and within-herd prevalence were both set to 30%, between 62 and 745 infected herds, with between 2,144 and 21,563 infected cattle, per TCZ were simulated. At this higher between-herd prevalence, between 10 and 11 herds per TCZ were required to be tested for case detection with both parallel and serial interpretation. Fewer TCZ simulations had zero infected herds sampled (a median of 2 simulations out of 100 replicates). No false positive or false negative cattle or herds were simulated. Apparent between-herd prevalence (based on median estimates from 100 simulations per TCZ) ranged from 20 to 36% (Fig. 6).

## Discussion

Our paper describes a potential scenario of a BT case detection in Ireland and subsequent surveillance to detect cases in a surrounding 20 km control zone. The BTV-8 outbreak in Europe between 2006 and 2009, and the more recent emergence of BTV-3 in northern Europe and England demonstrate that BTV can disperse widely [10, 11] but it is challenging to predict potential BTV-3 transmission patterns in Ireland, given that temperature, wind conditions, season, midge and virus ecology and livestock management practices will all affect potential conditions for spread [18, 19, 24, 37]. For example, midge and virus ecology may be affected by temperature and rainfall [7, 17, 19, 20, 24]. Irish livestock management practices include a relatively high proportion of grass-based production and cattle movements [22, 23]. Understanding the extent of transmission after an incursion will assist stakeholders’ decision making on control measures, and this requires effective surveillance.

This study demonstrates that surveillance to estimate local prevalence, with an assumed between-herd prevalence of 5% and within-herd prevalence of 30%, performs well when test specificity is perfect and test sensitivity is 99%, and when between 39 and 61 herds per TCZ (depending on total herds in the TCZ) are tested. Apparent herd and animal level prevalence from test results were close to the simulated true values. When a higher between-herd prevalence of 30% is assumed, which would be consistent with more extensive transmission, sampling between 10 and 11 herds per TCZ provided an adequate estimate of prevalence. Our simulation allowed exploration of planned surveillance using probabilistic methods, and demonstrated that, with 5% between-herd prevalence, no infected herds would be sampled in approximately 5% of the TCZ simulations. This reflects the random nature of sampling where, by chance, no infected herds are selected. This is not surprising given that infected herds are relatively rare (1 in 20). With 30% between-herd prevalence, approximately 2% the TCZ simulations had no infected herds sampled.

Our 5% and 30% between-herd prevalence assumptions are low compared to some other European contexts. We assumed these prevalences for our work because potential transmission patterns of BTV-3 in Ireland are unknown, and we wished to provide sample size estimates appropriate for the earlier stages of BTV spread, with relatively lower between-herd prevalence. Between-herd prevalence of BTV-3 increased above 60% in the Netherlands [3, 15] and for BTV-8 in parts of East Anglia in 2007 and 2008 [21].

We selected RT-PCR diagnostic characteristics for our main analysis, as EFSA suggests a detection window of 4–5 months for detection of BTV nucleic acid in cattle after infection [10] and it is a highly sensitive and specific assay [41]. Assuming perfect specificity of the RT-PCR meant no issues with false-positives. Our calculation of PPV with a lower diagnostic specificity, and simulation outputs presented in our Supplementary Materials, highlight potential issues with false positives in a low prevalence context and can be explored further using our web application. This can be used in the context of evolving prevalence and understanding of test characteristics. Diagnostic test results are rarely interpreted in isolation. For example, we must integrate consideration that an animal is likely to have BTV RNA detectable in their blood by RT-PCR in advance of generating antibodies against BTV. A positive serological result may reflect exposure to BTV or a related virus, or vaccination against BTV, at any time in the past [10]. Vaccination against BTV has never been implemented in Ireland. However, imported animals may have been vaccinated against or exposed to BTV. Consideration of the local epidemiological situation, including animals with clinical signs, is also necessary. Additionally, there may be increased confidence in a positive result within a cluster of positive results, compared to an isolated positive result in a cluster of negative results.

We explored the effects of assuming either lower or higher between-herd prevalence in our scenarios because Ireland has never had a BT outbreak, so transmission characteristics of BTV are unknown. Transmission suitability for BTV varies according to weather and environmental conditions [18, 19]. These impact both virus and vector ecology. *Culicoides* are poikilothermic, and the rate that BTV can replicate to transmissible levels within the midge after it is ingested increases with environmental temperature [25]. Midge abundance is also strongly impacted by environmental conditions. Möhlmann et al. [19] assessed midge catch counts from Italy, Sweden and the Netherlands in relation to BTV risk and reproductive ratio. They reported that midge catches were higher in farm habitats than in wetland or peri-urban habitats and increased with the previous week’s mean daily precipitation. Within wetlands, variation (in the previous 30 days) in precipitation was associated with higher midge catches (possibly associated with larval sites escaping flooding in these areas). In Italy, the Netherlands and Sweden, midge catches were highest at a temperature of 20–21 °C. Catches decreased with increasing mean wind velocity 24 h before collection. Midge catches also varied more with season in the Netherlands and Sweden than in Italy [19]. It is unknown how the ecology of the midge species in Ireland [16, 17] may differ from this. Experiences with SBV over the past decade show that a midge borne virus infecting livestock can establish itself in Ireland [5–7]. However, Gubbins et al. [24] argued that the more extensive spread of SBV compared to BTV in England was due to a higher probability of SBV transmission from host to vector and differing temperature requirements for virus replication, highlighting that virus and vector ecology, including vector competence under different conditions, play an important role in transmission dynamics.

Our study had limitations including the decision we made to target larger cattle herds for surveillance. We justify this by highlighting that cattle are at higher risk of infection compared to sheep [4, 10, 14]. We use our simulation to demonstrate that focusing on larger cattle herds has minimal impact on surveillance effectiveness in cattle. We also highlight that the highest cattle density and largest cattle herds are present in East and South Ireland, where previous midge borne Schmallenberg virus incursion and transmission occurred [6, 23]. In absence of data associated with BTV transmission in Ireland, we cannot fully predict spatial heterogeneities in transmission, for application to our surveillance plan. The focus on larger cattle herds may need to be adapted in the face of evidence that BT transmission patterns in Ireland differ from other contexts. However, BTV transmission ecology in European settings [24, 36, 37] as well as EU legislation [42], support our current approach of using 20 km radius TCZs as our geographical surveillance units. These units, informed by BTV epidemiology and ecology, may mitigate much of the potential geographic bias relating to sampling larger cattle herds.

We acknowledge that another susceptible species, sheep, may reside in geographically distinct areas (uplands) to large dairy herds. This issue is partially ameliorated as passive surveillance may be more effective in sheep due to more pronounced clinical signs and mortality [43]. Further, active surveillance of cattle in the likely areas of incursion and initial transmission may provide early warning and enhanced surveillance in sheep.

There is active research in Ireland to better understand vector distribution [44], and evidence is also emerging on BTV-3 transmission patterns in England [45], which may have relatively more ecology and weather in common with Ireland compared to further afield in Europe. Better understanding of potential transmission biology of BTV-3 in Ireland would facilitate the development of a dynamic BTV-3 transmission model. This may offer a more nuanced understanding of disease dynamics and potential effectiveness of interventions, compared to our straightforward Monte Carlo simulations, assuming static prevalence. Enhanced understanding of transmission biology may also be incorporated into our online application in the future.

Uncertainties about the potential transmission patterns of BTV in Ireland highlight the importance of effective surveillance after an incursion to detect additional cases. For example, if we are confident that prevalence is low and BTV is not spreading extensively, testing and culling may be a feasible control mechanism. In contrast, mass vaccination, as is currently used in several countries to reduce BTV-3 transmission and impacts, may be more appropriate if BTV reaches a high prevalence. The evolution of European legislation on BTV controls reflects increasing incursions of BTV into, and, in some cases, becoming established in, many EU countries. There is recognition that aggressive “stamping out” based eradication policies may not be possible in all cases. The Animal Health Law currently defines BT as a Category C disease allowing countries to engage in optional control programmes, in contrast to its previous status as a “Class A” disease requiring strict controls described by the old Council Directive 2000/75/EC, which has been superseded by the Animal Health Law.

In whichever way the potential transmission patterns and policy context may evolve, case detection to better understand the extent of BTV spread after a known introduction will help inform the Irish response to an incursion. Our simulation study provides evidence to inform the Irish programme for detection of potential incursion and spread of BTV-3.

## Supplementary Information

Supplementary Material 1.

## Data Availability

The datasets analysed during this study are available from the Department of Agriculture, Food and the Marine (DAFM), and summarise for each TCZ and be viewed on our web application. The full data are subject to data protection regulations and limitations ([https://www.gov.ie/en/organisation-information/ef9f6-data-protection/] (https://www.gov.ie/en/organisation-information/ef9f6-data-protection). All R code for the simulation and an example dataset are available here: [https://www.github.com/miriamcasey/BTVsurveillance].

## Footnotes

1
**Innovative Diagnostics**, 310 rue Louis Pasteur – 34,790 Grabels, France.
