# Supplementary material for: Preparation for a potential outbreak of bluetongue virus in Ireland: surveillance design to estimate local prevalence after an initial case detection
Source: Ir Vet J. 2025 Nov 20;78:30. doi: 10.1186/s13620-025-00315-1 (PMC12631999; doi:10.1186/s13620-025-00315-1)
Supplement: Supplementary file 1 — Supplementary Material 1. [file 13620_2025_315_MOESM1_ESM.docx]

Preparation for a potential outbreak of bluetongue virus in Ireland: surveillance design to estimate local prevalence after an initial case detection

Supplementary materials

Miriam Casey-Bryars^1,2^, Jamie A. Tratalos^1^, Jamie M. Madden^1^, Guy McGrath^1^

1. Centre for Veterinary Epidemiology and Risk Analysis, School of Veterinary Medicine, University College Dublin, Belfield, Dublin 4, Ireland.
2. School of Veterinary Medicine, University College Dublin, Belfield, Dublin 4, Ireland.

Correspondence to: Miriam Casey-Bryars; [miriam.casey@ucd.ie](mailto:miriam.casey@ucd.ie), School of Veterinary Medicine, University College Dublin, Belfield, Dublin D04 W6F6, Ireland.

Contents

[Data from English outbreak of BTV-8 in 2007 and 2008 2](#_Toc206161596)

[Data from the Dutch outbreak of BTV-3 in 2023 and 2024 3](#_Toc206161597)

[Distribution of cattle, sheep and early stage Schallenberg virus cases in Ireland 5](#_Toc206161598)

[Supplementary results for a scenario with 99% test specificity and perfect test sensitivity 9](#_Toc206161599)

[Positive and negative predictive values with 100% sensitivity and 99% specificity. 9](#_Toc206161600)

[Simulation results with 5% between-herd, 30% within herd prevalence, 100% sensitivity and 99% specificity. 10](#_Toc206161601)

[Simulation results with 30% between-herd, 30% within herd prevalence, 100% sensitivity and 99% specificity. 11](#_Toc206161602)

[References for supplementary material 13](#_Toc206161603)

## Data from English outbreak of BTV-8 in 2007 and 2008

We used BTV-8 surveillance data from England in 2007 and 2008 (1), comprising targeted surveillance around confirmed cases. Surveillance was reported in Ipswich, Lowestoft and Peterborough in East Anglia in September 2007. Ipswich and Lowestoft were close to the initial incursion site, whereas Peterborough was further west. There were also data from Poole based on follow up surveillance after an initial confirmation from a pre-movement test in February 2008. Ipswich and Lowestoft had between-herd prevalences of 61% and 66%, respectively, whereas targeted testing in Peterborough did not detect any further cases. In Poole, a five percent between herd prevalence was detected. The same English BTV-8 dataset reported within herd prevalence, in 39 herds, of between 1% and 51% with a median of 6% and a mean of 11%.

## Data from the Dutch outbreak of BTV-3 in 2023 and 2024

In a longitudinal study of 5 cattle herds with BTV-3 in the Netherlands in October 2023 to February 2024 (2), PCR positivity rates ranged from 5 - 51% (mean = 32.2%) in the first week of the study, and seropositivity ranged from 5 – 41% (mean = 16%). In the thirteenth week of the study seropositivity ranged from 4% - 55% (mean = 26%). Within-herd prevalence and morbidity (the proportion of animals with reported clinical signs) was higher in cattle than in sheep herds. However, case fatality and overall change in mortality compared to the previous year was markedly higher in the sheep herds. Reports from the Netherlands suggest that there are more severe clinical signs associated with BTV-3 than with BTV-8.

A between-herd prevalence study was conducted on unvaccinated Dutch dairy herds using bulk milk ELISA testing for antibodies against BTV in Autumn 2023. Overall between-herd prevalence was 64% (95% CI: 63 – 65%), ranging from a minimum of five percent in the least affected region to 99% in the most heavily infected. Although this methodology is not yet described or peer-reviewed, based on semiquantitative estimates from the milk ELISA, Dutch experts estimated that within-herd prevalence was 36% (95% CI: 35 – 37%), ranging from 13% in the least affected area to 56% in the most affected (3) . Without considering herd-level clustering, the same study estimated animal level prevalence of 23% (95% CI: 22 – 24%), ranging from 1% to 51% depending on the region.

**Supplementary table 1: Within herd morbidity, mortality, PCR and seropositivity data extracted from a study of a sample of herds with at least 100 animals in the Netherlands which had BTV-3 infections confirmed in October 2023 and which were monitored over a 13 week period until February2024** (2)**. The percentages in brackets represent the minimum and maximum for each study group). * Where figures were not reported in the text of the paper they were estimated from plots.**

| **Species** | **N farms** | **Morbidity**  **(%)** | **Mortality** | **PCR positive rate week 1***  **(%)** | **Seropositivity week 1***  **(%)** | **Seropositivity week 13**  **(%)** |
| --- | --- | --- | --- | --- | --- | --- |
| Cattle | 5 | 24.5 (8.1 – 50.4) | Low. 1 cow reported dead in 4 of 5 farms versus 0 previous year. | 23.2 (5 – 51) | 16 (5 – 41) | 26 (4 - 55) |
| Sheep | 5 | 7.5 (0.9 – 14.2) | High. Case fatality rate was 74.8. On average 45 sheep per herd dies in study period compared to 13 previous year. | 13 ( 2 – 30) | 12 (2 – 22) | 7 (2 – 22) |
| Goats | 3 | 1.1 (0.3 – 3.6) | Case fatality 44. Mortality not different to previous year. |  |  | (0 – 12) |

## Distribution of cattle, sheep and early stage Schallenberg virus cases in Ireland


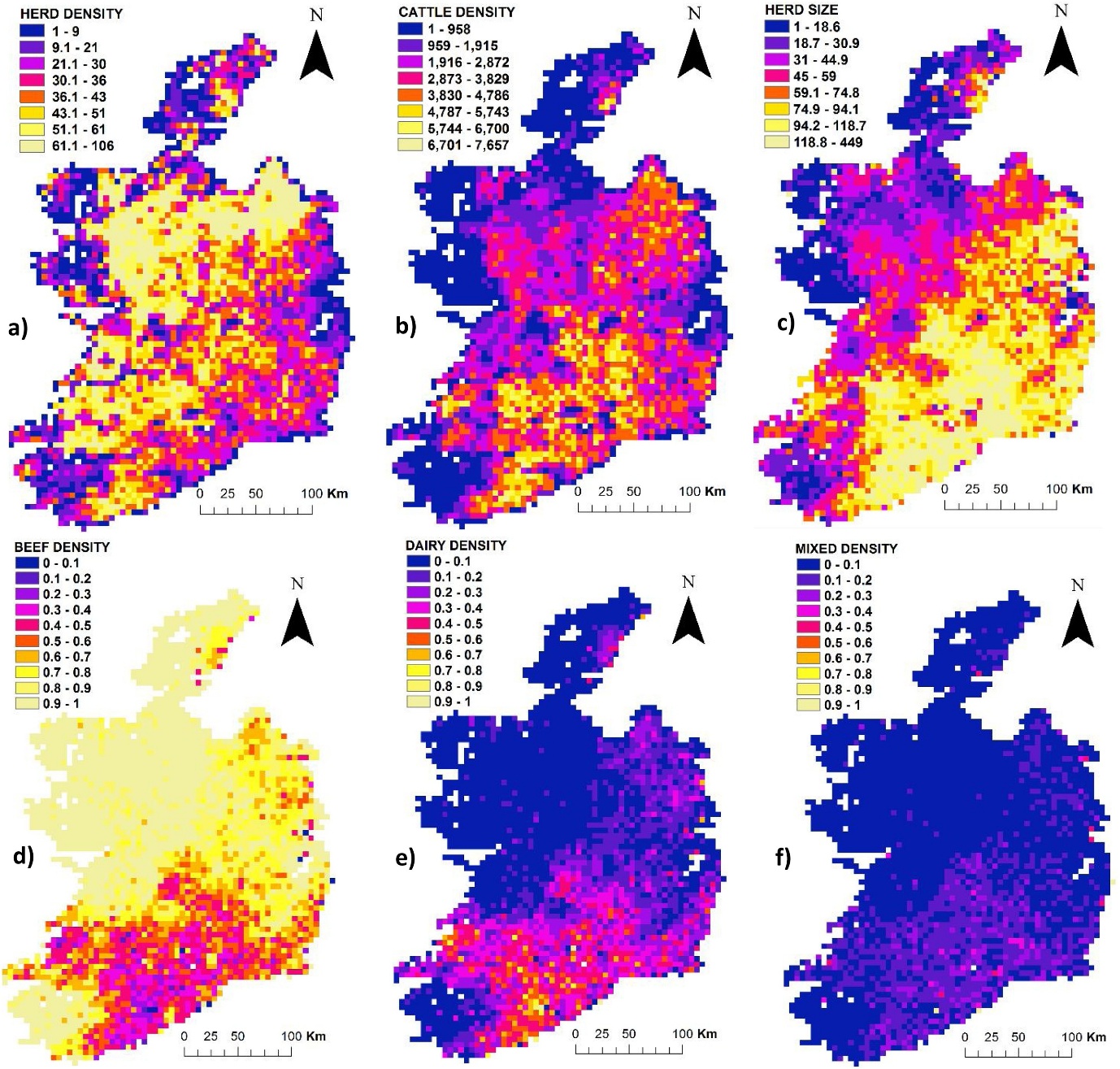


**Supplementary Figure 1 from Tratalos et al 2020** (4)**:  Six metrics showing the distribution of the cattle population of Ireland, displayed on a 5 km grid: a) the number of herds, b) the number of cattle (all bovines), c) mean herd size, d-f) the proportion of herd consisting of beef, dairy or mixed herd types. Calculated for 30 June 2016.**

**
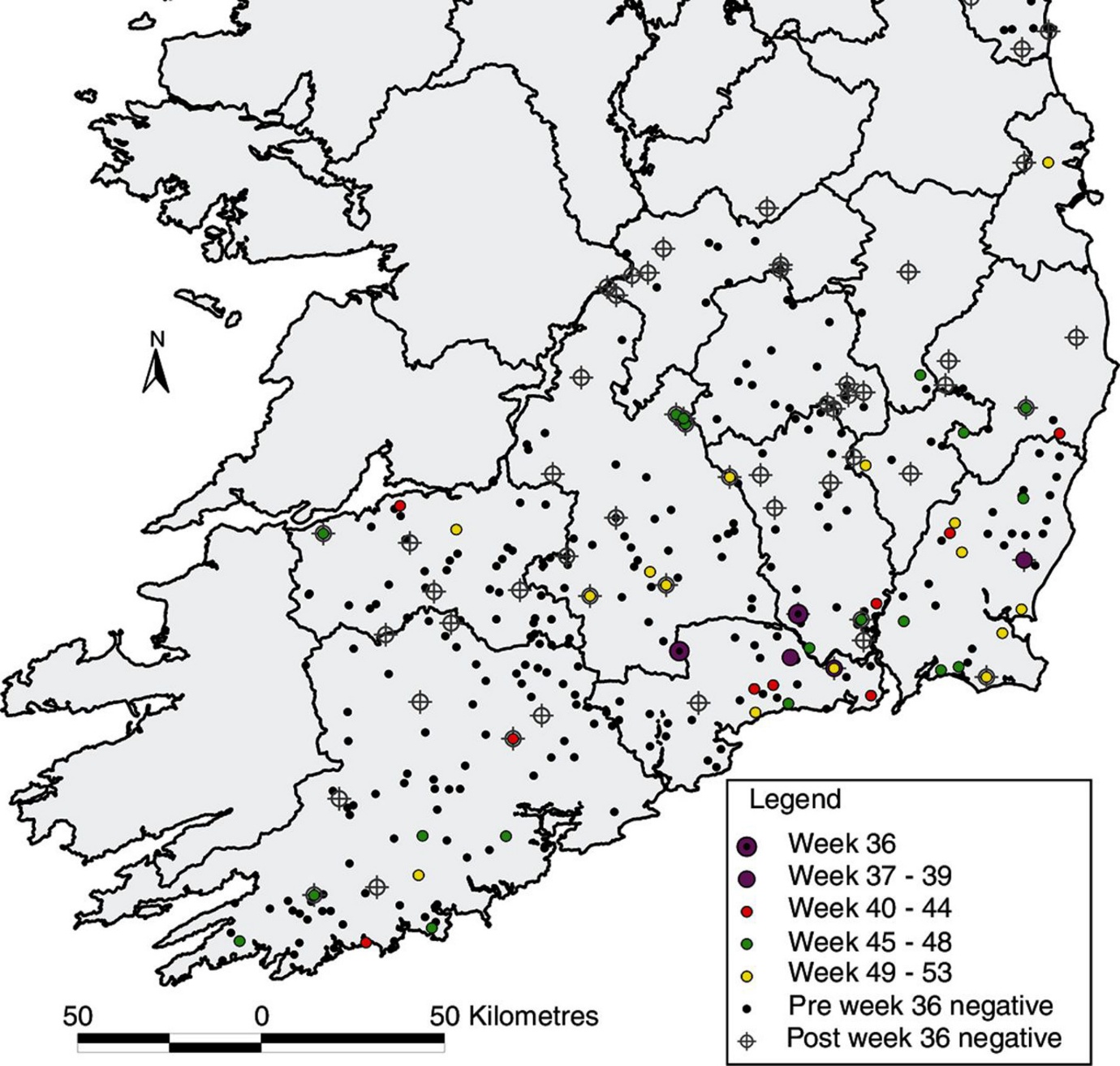
**

**Supplementary figure 2 adapted from McGrath et al 2018** (5)**: The progression of exposures to Schmallenberg virus in south-east Ireland during March to December 2012.**

**
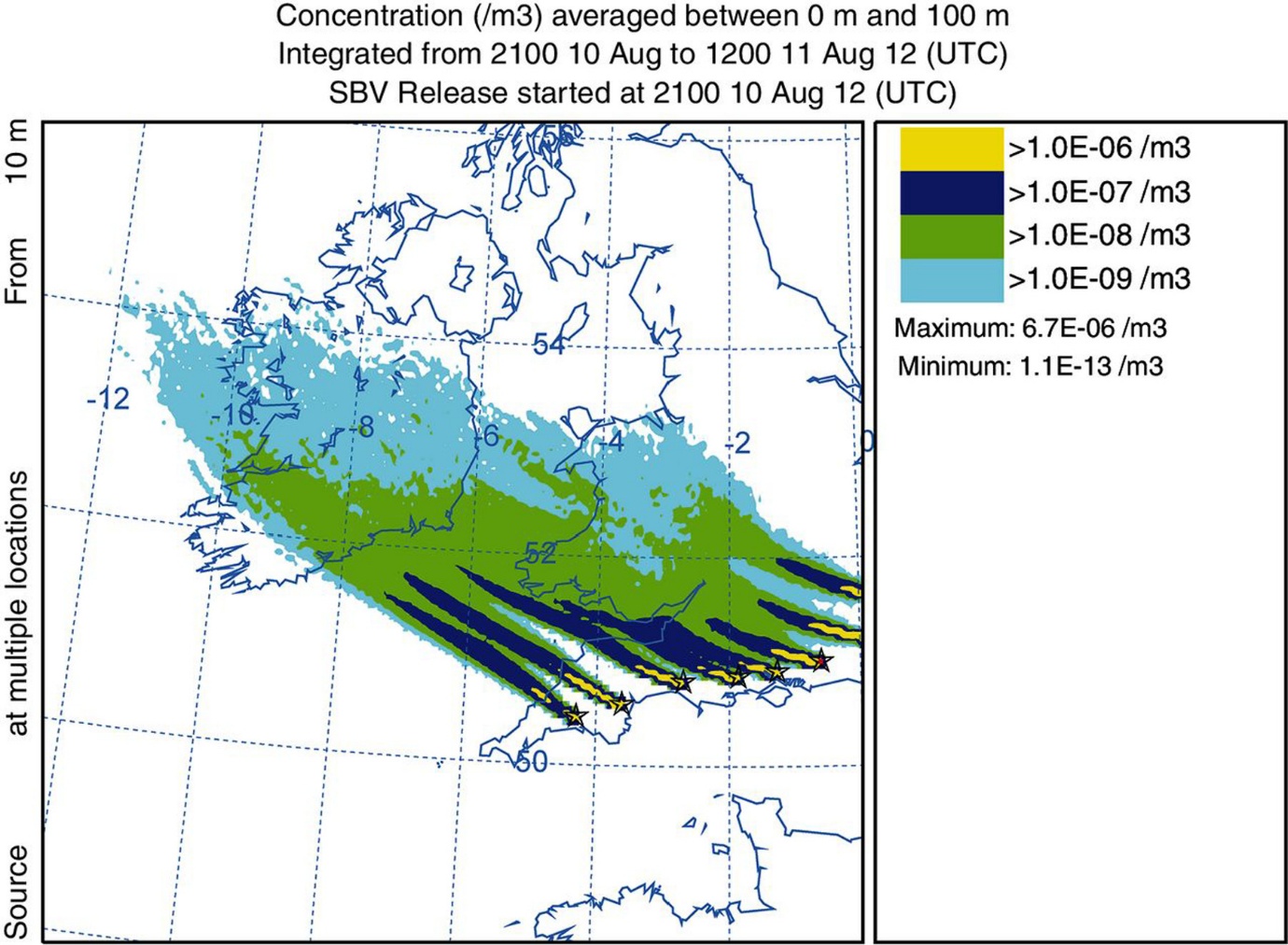
**

**Supplementary figure 3 adapted from McGrath et al 2018** (5)**: Particle concentration model assuming a continuous release from source locations starting at 21:00 on August 10, 2012 until 12:00 on August 11, 2012. Concentration values calculated from an arbitrary input of 100,000 released particles from each source location over a 15-hour period. Time in Coordinated Universal Time (UTC).**


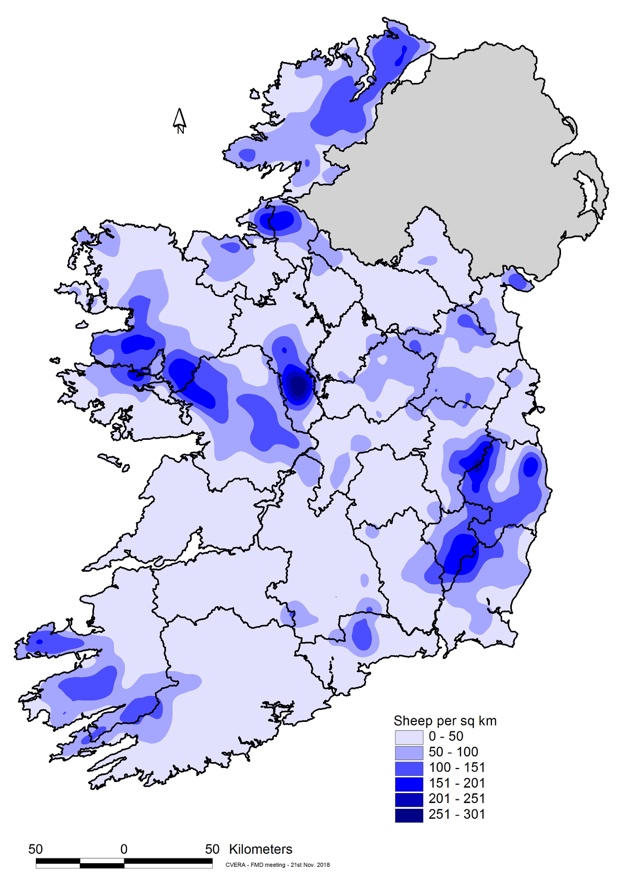


**Supplementary Figure 4: Density of sheep in Ireland based on the Sheep population census from 2016**(6)**.**

## Supplementary results for a scenario with 99% test specificity and perfect test sensitivity

### Positive and negative predictive values with 100% sensitivity and 99% specificity.


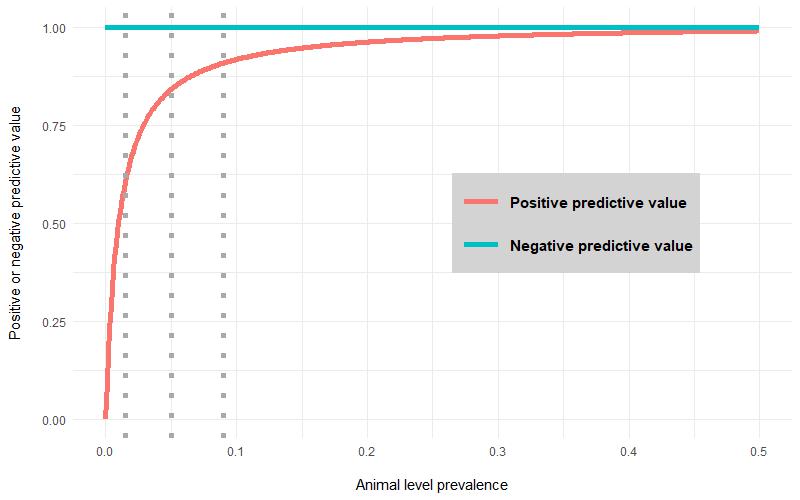


**Supplementary Figure 5: Based on a test specificity of 99% and sensitivity of 100%, variation in positive and negative predictive values with overall animal level prevalence. The vertical dotted lines represent animal level prevalence of 1.5% (corresponding for example to a between herd prevalence of 5% and a within-herd prevalence of 30%), 5 % animal-level prevalence (EU guideline) and 9% animal level prevalence (corresponding for example to a between herd prevalence of 30% and a within-herd prevalence of 30%).**

### Simulation results with 5% between-herd, 30% within herd prevalence, 100% sensitivity and 99% specificity.

Amongst the cattle selected for testing, median counts of test positive infected cattle (true positives) in each TCZ ranged from 4 to 11 per TCZ and counts of test negative uninfected cattle (true negatives) ranged from 381 to 597. False positive animals in the TCZ ranged from 3 - 7 and , as sensitivity was 100%, there were no false negatives. Within test positive herds, mean apparent animal level prevalence ranged from 15 - 20 per cent (as there were lower counts of positives in false positive herds) . Apparent herd level prevalence was overestimated at between 11 per cent and 16 per cent (Supplementary Figure 6). True positive herds ranged from 1 to 3. Uninfected herds incorrectly diagnosed as positive ranged from 3 – 6.

**
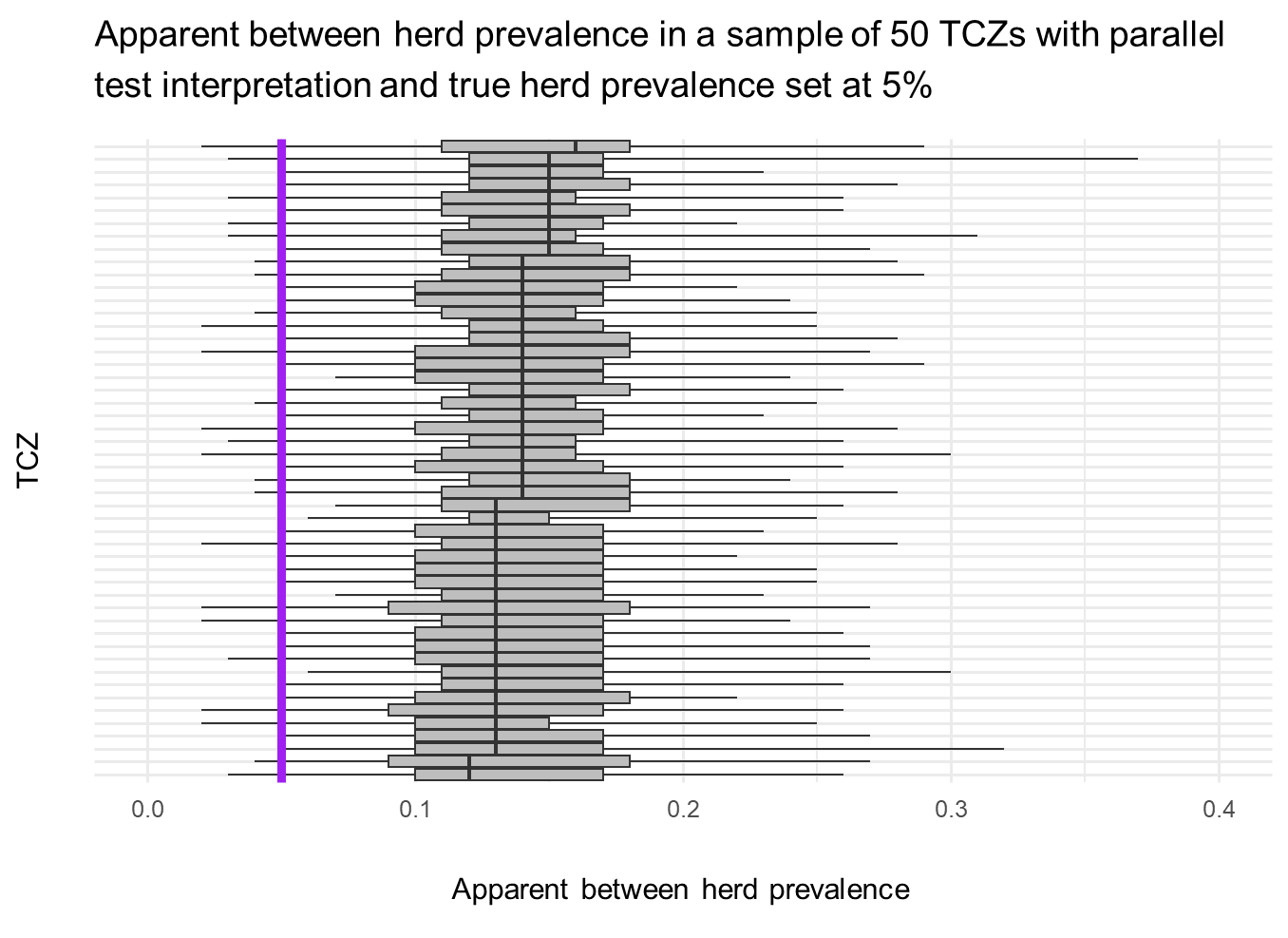
**

**Supplementary Figure 6: Apparent between herd prevalence in 50 randomly selected temporary control zones (TCZs) test sensitivity of 100%, test specificity of 99% and simulated true between herd prevalence of 5% (purple line). The end of the whiskers represent the maximum and minimum, the box represents the IQR and the vertical line is the median. The plot is ordered by median apparent herd prevalence. A sample of TCZs only are shown to improve plot clarity.**

### Simulation results with 30% between-herd, 30% within herd prevalence, 100% sensitivity and 99% specificity.

False positive results were less of an issue when a higher between-herd prevalence of 30% was simulated. No TCZ simulation had greater than a single false positive bovine and a single false positive herd. Of the 10 – 11 herds in each TCZ tested, count of true positive herds ranged from 2 – 4. Proportion of herds testing positive ranged from 27 per cent to 41 per cent and slightly overestimated true between-herd prevalence (Supplementary Figure 7).


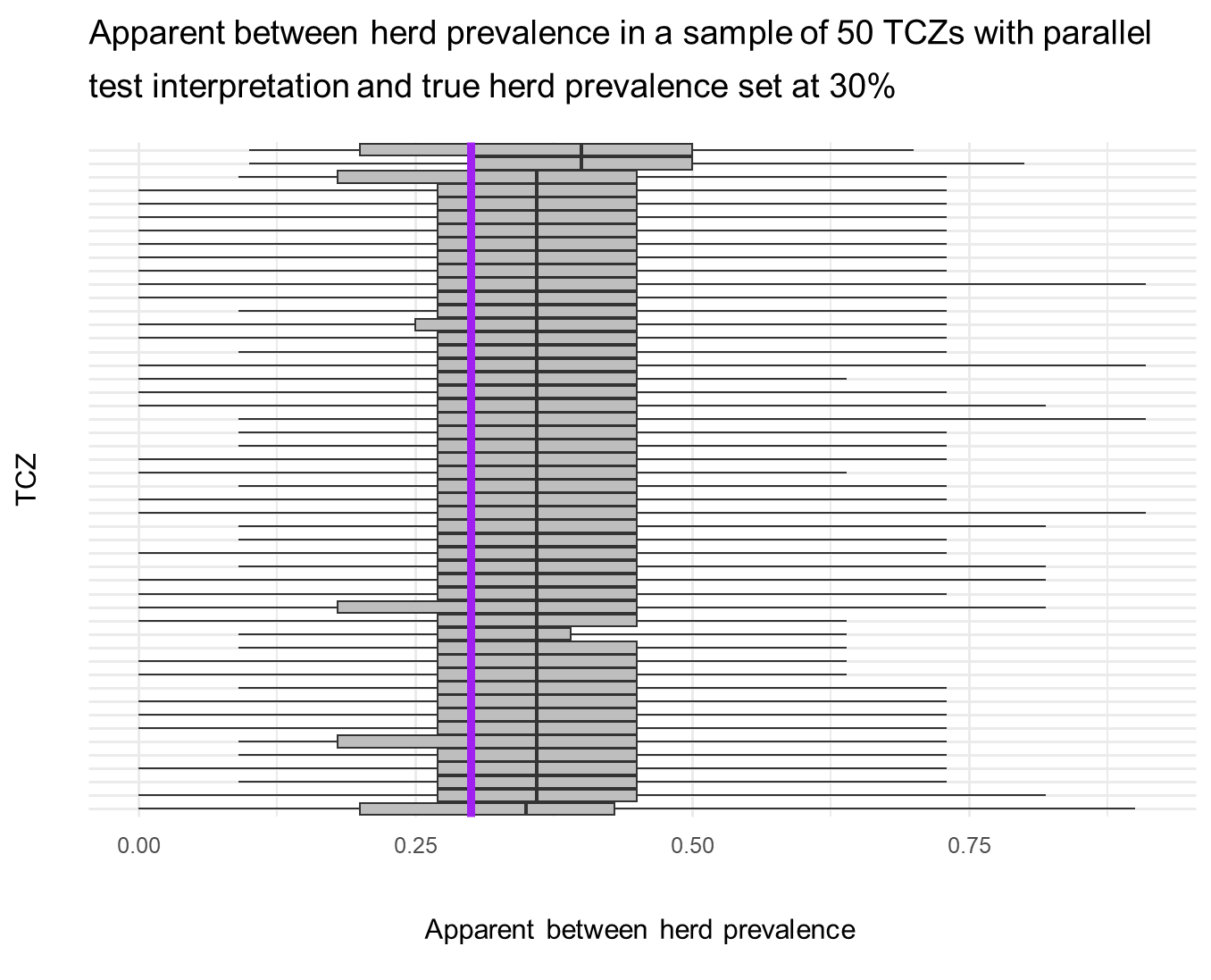


**Supplementary Figure 7: Apparent between herd prevalence in 50 randomly selected temporary control zones (TCZs) level with parallel test interpretation and simulated true between herd prevalence of thirty per cent (purple line). The end of the whiskers represent the maximum and minimum, the box represents the IQR and the vertical line is the median. The plot is ordered by median apparent herd prevalence. A sample of TCZs only are shown to improve plot clarity.**

# References for supplementary material

1. DEFRA. Food and Farming Group Veterinary Sciences Core team. 2008 [cited 2024 Sep 5]. p. 1–31 Report on the distribution of bluetongue infection in Great Britain on 15 March 2008. Available. Available from: https://webarchive.nationalarchives.gov.uk/ukgwa/20090731155903/http:/www.defra.gov.uk/animalh/diseases/notifiable/bluetongue/pdf/epi-report080508.pdf

2. Van den Brink KMJA, Santman-Berends IMGA, Harkema L, Scherpenzeel CGM, Dijkstra E, Bisschop PIH, et al. Bluetongue virus serotype 3 in the Netherlands; clinical signs, seroprevalences and pathological findings in ruminants. Veterinary Record. 2024;Accepted(July):1–10.

3. Santman-Berends I, van den Brink K, Mars J, Veldhuis A, Bogt-Kappert C ter. Royal GD Netherlands. 2024 [cited 2024 Sep 6]. Prevalence of bluetongue virus serotype 3 in Dutch cattle population. Available from: https://www.gddiergezondheid.nl/Diergezondheid/Onderzoek/Onderzoek-antistoffen-blauwtongvirus

4. Tratalos J, Madden J, McGrath G, Graham D, Áine Collins, More S. Spatial and network characteristics of Irish cattle movements. Preventative Veterinary Medicine. 2020;183.

5. McGrath G, More SJ, O’Neill R. Hypothetical route of the introduction of Schmallenberg virus into Ireland using two complementary analyses. Veterinary Record. 2018;182(8):226.

6. DAFM. National Sheep and Goat Census. 2018;(December).
